# Supplementary material for: Observation of quadratic Weyl points and double-helicoid arcs
Source: Nat Commun. 2020 Apr 14;11:1820. doi: 10.1038/s41467-020-15825-5 (PMC7156696; doi:10.1038/s41467-020-15825-5)
Supplement: Supplementary file 1 — Supplementary Information [file 41467_2020_15825_MOESM1_ESM.pdf]

Supplementary Information

Observation of quadratic Weyl points and double helicoid arcs, He et al.

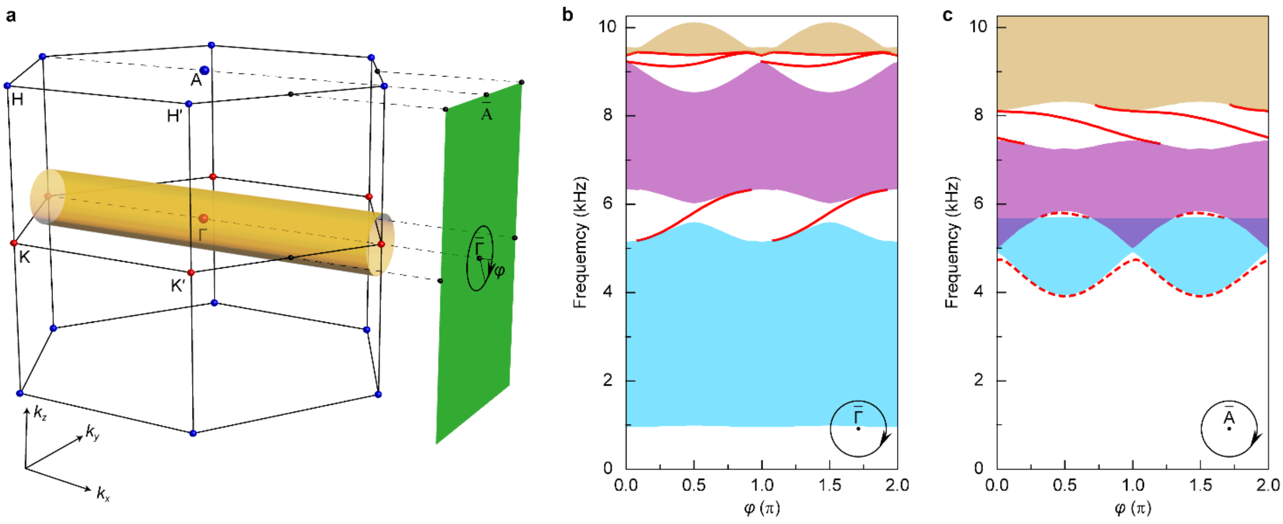

Supplementary Figure 1 | Surface spectra calculated for the YZ surface. a, Bulk BZ and its surface projection to the k_y-k_z plane. **b**, Projected band structure simulated along the circular path (of radius $0.2\pi/a$) centered at $\bar{\Gamma}$. The two gapless surface bands (red lines) that link the lowest and middle projected bands come from the two time-reversal-related CWPs at K and K′, and the two gapless surface bands connecting the middle and the highest projected bands stem from the QWP at Γ. The numbers and overall slopes of the surface bands reflect precisely the information of the topological charges. **c**, Similar to **b** but for a path centered at $\bar{\mathrm{A}}$. The two gapless surface bands connecting the middle and the highest projected bands come from the two CWPs at H and H′. Similar to Fig. 3d, no topological surface band emerges between the lowest two bulk bands since there is no gap. The red dashed lines correspond to topologically trivial surface states. (Note that the CWPs at K and K′ with identical charges are projected simultaneously onto the $\bar{\Gamma}$ point of the k_y-k_z surface BZ, and overlapped with the projection of the QWP at Γ. The surface states emanating from the two CWPs and the QWP are distinguished in frequency, which contains the information of topological charges for each Weyl nodes. Similarly, both the CWPs at H and H′ are projected onto $\bar{\mathrm{A}}$ and overlapped with the surface projection of the QWP at A.)

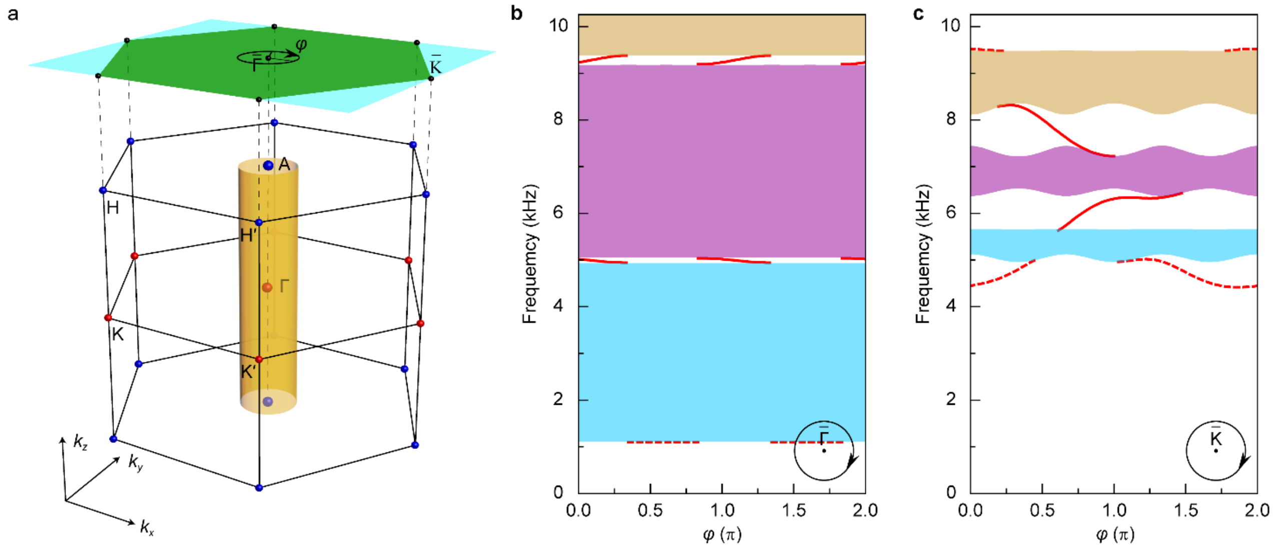

Supplementary Figure 2 | Surface spectra calculated for the XY surface. a, Bulk BZ and its surface projection to the k_x-k_y plane. Note that the surface projections of the QWPs at Γ and A overlap at $\bar{\Gamma}$, and that the surface projections of the CWPs at K (K′) and H (H′) overlap at $\bar{K}$ ($\bar{K}'$). **b**, Projected band structure simulated along the circular path (of radius $0.2\pi/a$) centered at $\bar{\Gamma}$. The two gapless surface bands (red lines) that link the lowest and middle projected bands come from the QWP at A, while the two gapless surface bands connecting the middle and the highest projected bands stem from the QWP at Γ. The numbers and overall slopes of the surface bands reflect precisely the information of the topological charges. **c**, Similar to **b** but for a path centered at $\bar{K}$. The gapless surface band connecting the lowest and the middle projected bands come from the CWP at K, and the gapless surface band connecting the middle and the highest projected bands come from the CWP at H. The red dashed lines correspond to topologically trivial surface states.

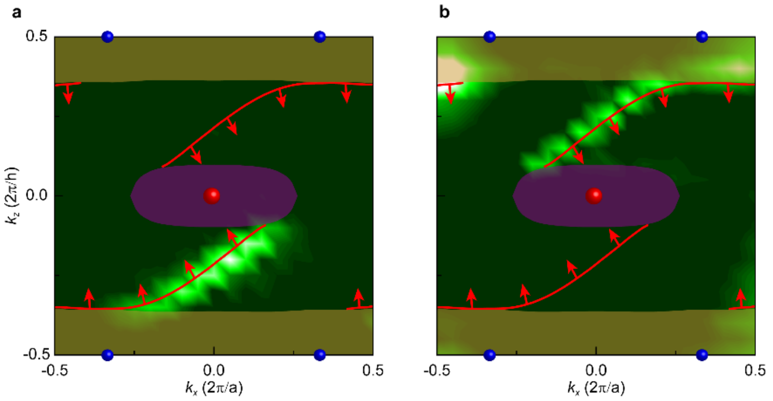

Supplementary Figure 3 | Surface arc excited by a single point source positioned at the corner of the sample. **a**, 2D Fourier transformation (color scale) of the surface field excited by a point source positioned at the bottom right corner of the sample at 8.64 kHz, compared with the numerical isofrequency contours of the topological surface states (red lines). As before, the color spheres highlight the projected Weyl nodes, and the shaded areas are the projected bulk bands. **b**, The same as **a**, but for the source positioned at the upper left corner. As expected, in each case the point source excites only one of the surface arcs according to the group velocities (labeled with arrows) of the surface states.

Note: We use these two independent point sources to attain the surface spectrum in the main text. Technically, we summed the pressure fields and then performed Fourier transform considering the fact that each source excites only one surface arc. By doing so, we can

(i) Reduce the finite-size effect. We locate the point source at the corner of the sample to effectively maximize the propagation distance of specific surface states, which improve the momentum resolution of Fourier spectrum.

(ii) Improve the excitation efficiency of surface states. The above treatment, instead of locating the source at the sample center, reduces the excitation efficiency of the surface states. This is improved by locating two independent point sources at different sample corners, each igniting one surface arc according to the known group velocity.

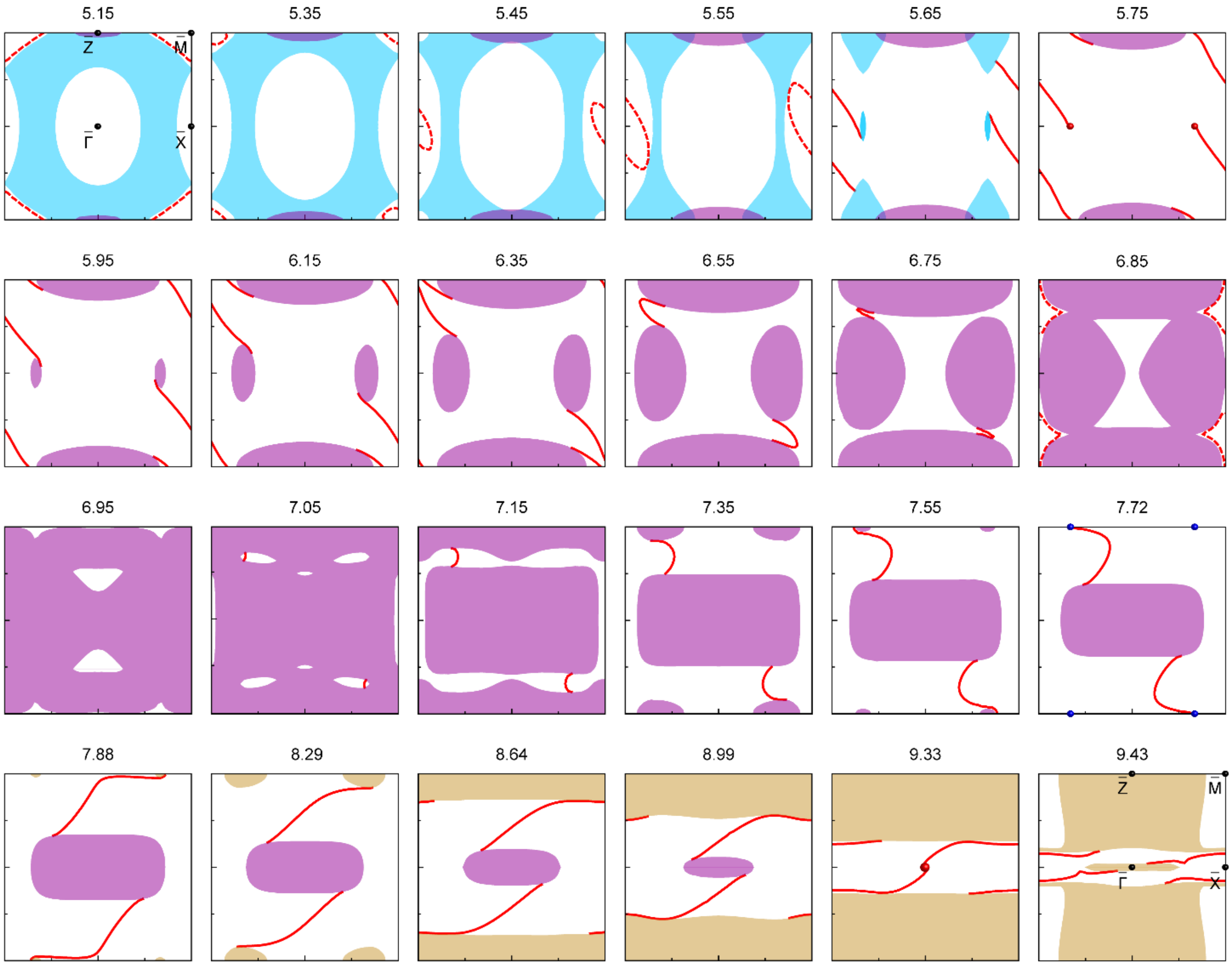

Supplementary Figure 4 | Broadband isofrequency contours for the XZ surface (in unit of kHz). As before, the colored regions indicate the projections of the lowest three bulk bands. As a supplementary for the simulation results of Fig. 4b in main text, which involve only the middle and the highest bulk bands, the figures here provide an evolution of the topological surface arcs over a broader range of frequencies (red solid lines). The red dashed lines, which form *closed* loops across the surface BZ, correspond to topologically trivial surface states.

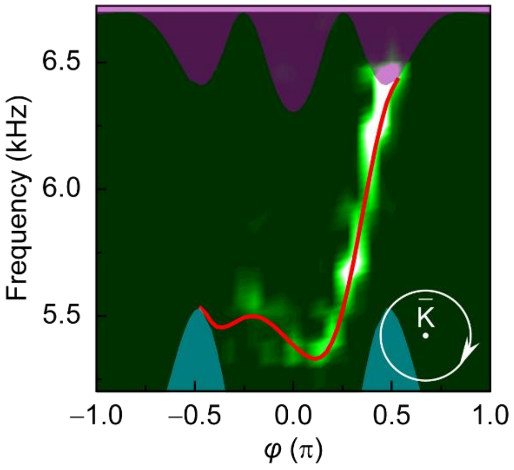

Supplementary Figure 5 | Measured XZ surface spectrum along a loop centered at $\bar{\mathrm{K}}$. The loop has a radius of $0.43\pi/a$ and encircles only the projected lower-frequency CWP at K. In accordance with the simulation result, it shows only one topological surface band traversing the projected gap between the lowest and the middle bulk bands, exhibiting an overall negative slope inside the gap. In this measurement, we consider two independent excitations on the up-right and down-left corners of the XZ surface, given the group velocity information of the topological surface states in this case.

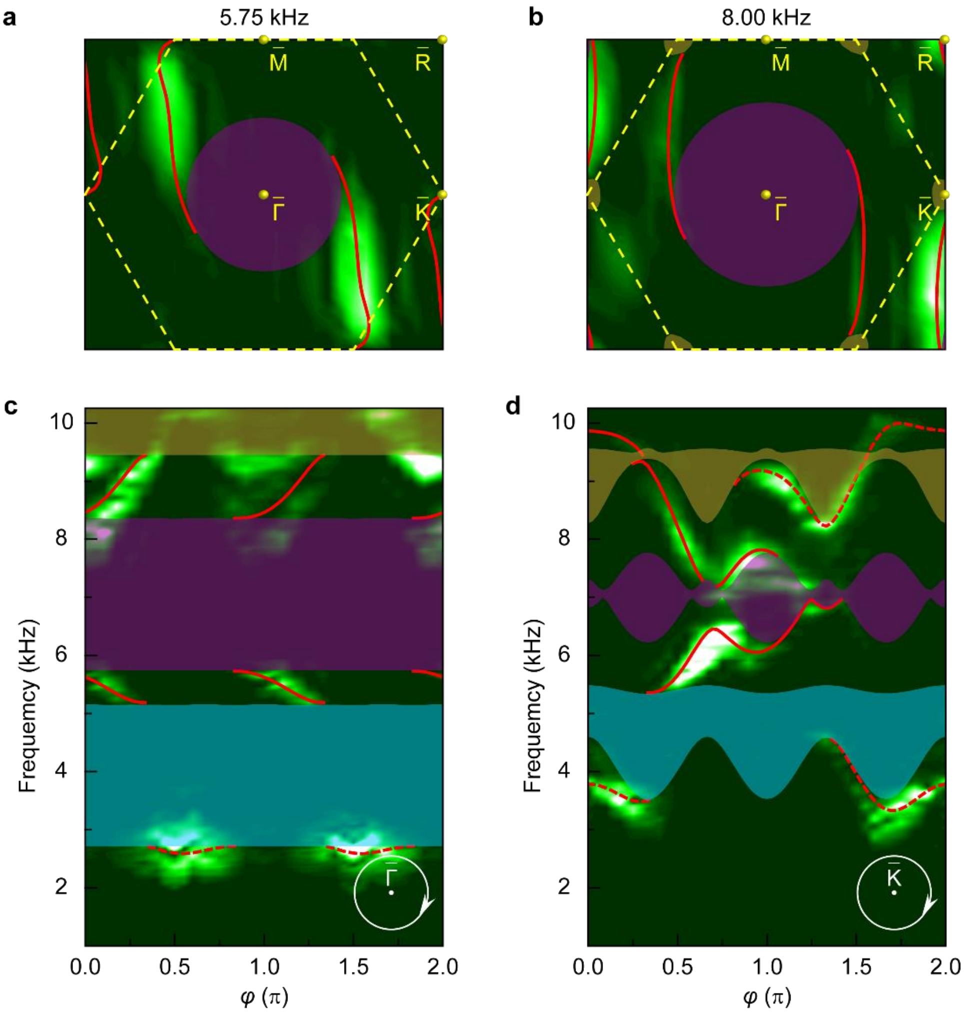

Supplementary Figure 6 | Experimental identification of the surface states at the XY surface. The main features of the simulations (red lines) are captured by our experiments (color scale). **a** and **b**: Surface arcs exemplified at 5.75 kHz and 8.0 kHz, which are very close to the frequencies of the CWPs at K (K′) and H (H′), respectively. At 5.75 kHz, two surface arcs emitting separately from $\overline{\mathrm{K}}$ and $\overline{\mathrm{K}}'$ are well excited in the first surface BZ and terminated at the DWP pocket centered at $\overline{\Gamma}$. At the higher frequency, 8.0 kHz, weakly excited surface arcs that link $\overline{\mathrm{K}}$ ($\overline{\mathrm{K}}'$) and $\overline{\Gamma}$ pockets can be observed in the first surface BZ, together with strong excitations in the second surface BZ. **c**, Surface spectrum extracted along a circular path (of radius $0.5\pi/a$) centered at $\overline{\Gamma}$. Two gapless topological surface states emerge in each band gap formed between the lowest three bulk bands. **d**, Similar to **c** but for a loop centered at $\overline{\mathrm{K}}$. As expected, only one gapless topological surface state appears inside each band gap.
